# Restoration of aberrant gene expression of monocytes in systemic lupus erythematosus via a combined transcriptome-reversal and network-based drug repurposing strategy

**DOI:** 10.1186/s12864-023-09275-8

**Published:** 2023-04-18

**Authors:** Dimitrios Nikolakis, Panagiotis Garantziotis, George Sentis, Antonis Fanouriakis, George Bertsias, Eleni Frangou, Dionysis Nikolopoulos, Aggelos Banos, Dimitrios T Boumpas

**Affiliations:** 1 Amsterdam Institute for Gastroenterology Endocrinology and Metabolism, Department of Gastroenterology, Amsterdam UMC, Academic Medical Center, University of Amsterdam, Meibergdreef 9, Amsterdam, 1105 AZ The Netherlands; 2 Department of Rheumatology and Clinical Immunology, Amsterdam Rheumatology & Immunology Center (ARC), Amsterdam UMC, University of Amsterdam, Amsterdam, The Netherlands; 3 Amsterdam Institute for Infection & Immunity, Department of Experimental Immunology, Amsterdam UMC, University of Amsterdam, Amsterdam, The Netherlands; 4 Laboratory of Autoimmunity and Inflammation, Center for Clinical, Experimental Surgery and Translational Research, Biomedical Research Foundation of the Academy of Athens, Athens, Greece; 5 Department Rheumatology and Immunology, Hannover Medical School, Hannover, Germany; 6 Rheumatology and Clinical Immunology Unit, Department of Internal Medicine, Attikon University Hospital, Athens, 4th Greece; 7 Department of Propaedeutic Internal Medicine, “Laiko” General Hospital, Athens, Greece; 8 Joint Academic Rheumatology Program, National and Kapodistrian University of Athens Medical School, Athens, Greece; 9 Onassis Foundation, Athens, Greece; 10 Department of Rheumatology and Clinical Immunology, Medical School, University Hospital of Heraklion, University of Crete, Heraklion, Greece; 11 Institute of Molecular Biology and Biotechnology-FORTH, Heraklion, Greece; 12 Department of Nephrology, Limassol General Hospital, Limassol, Cyprus; 13 Medical School, University of Nicosia, Nicosia, Cyprus

**Keywords:** Systemic lupus erythematosus, Monocytes, Drug repurposing, Micro RNAs, Transcription factors

## Abstract

**Background:**

Monocytes -key regulators of the innate immune response- are actively involved in the pathogenesis of systemic lupus erythematosus (SLE). We sought to identify novel compounds that might serve as monocyte-directed targeted therapies in SLE.

**Results:**

We performed mRNA sequencing in monocytes from 15 patients with active SLE and 10 healthy individuals. Disease activity was assessed with the Systemic Lupus Erythematosus Disease Activity Index 2000 (SLEDAI-2 K). Leveraging the drug repurposing platforms iLINCS, CLUE and L1000CDS^2^, we identified perturbagens capable of reversing the SLE monocyte signature. We identified transcription factors and microRNAs (miRNAs) that regulate the transcriptome of SLE monocytes, using the TRRUST and miRWalk databases, respectively. A gene regulatory network, integrating implicated transcription factors and miRNAs was constructed, and drugs targeting central components of the network were retrieved from the DGIDb database. Inhibitors of the NF-κB pathway, compounds targeting the heat shock protein 90 (HSP90), as well as a small molecule disrupting the Pim-1/NFATc1/NLRP3 signaling axis were predicted to efficiently counteract the aberrant monocyte gene signature in SLE. An additional analysis was conducted, to enhance the specificity of our drug repurposing approach on monocytes, using the iLINCS, CLUE and L1000CDS^2^ platforms on publicly available datasets from circulating B-lymphocytes, CD4^+^ and CD8^+^ T-cells, derived from SLE patients. Through this approach we identified, small molecule compounds, that could potentially affect more selectively the transcriptome of SLE monocytes, such as, certain NF-κB pathway inhibitors, Pim-1 and SYK kinase inhibitors. Furthermore, according to our network-based drug repurposing approach, an IL-12/23 inhibitor and an EGFR inhibitor may represent potential drug candidates in SLE.

**Conclusions:**

Application of two independent - a transcriptome-reversal and a network-based -drug repurposing strategies uncovered novel agents that might remedy transcriptional disturbances of monocytes in SLE.

**Supplementary Information:**

The online version contains supplementary material available at 10.1186/s12864-023-09275-8.

## Background

Monocytes and macrophages constitute a major cellular compartment derived from hematopoietic myeloid precursors. Monocyte-macrophage lineage cells exhibit versatile immunoregulatory, inflammatory and tissue repairing capabilities and play an instrumental role in the development of systemic lupus erythematosus (SLE) [1]. Data from murine and human SLE studies demonstrated that the polyclonal B cell hyperreactivity, an immunological hallmark of SLE, might be at least partially attributable to aberrations in monocyte-mediated CD40/CD40L co-stimulation [1–5]. Abnormal activation of autoreactive T and B cells in SLE could also be caused by deregulated cytokine production by monocytes. Monocytes in SLE display excess production of the B-lymphocyte stimulator (BLyS) which promotes the survival and proliferation of B cells [6]. Moreover, these cells are a major source of IL-10 and IL-6 in the peripheral blood of SLE patients, which in turn augments antibody production and induces plasma cell differentiation, respectively. Despite its anti-inflammatory role in general, IL-10 derived from monocytes in SLE, can promote the production of the BLyS factor from B-lymphocytes, which is also linked with the development of autoantibodies [6]. Besides their contribution to the aberrant activation of adaptive immune system, defects in non-inflammatory phagocytosis by macrophages are implicated in the impaired clearance of cellular debris, that serves as a crucial trigger for the production of autoantibodies in SLE [1, 7–10]. Notably, monocytes in SLE not only significantly contribute to the generation of the interferon (IFN) signature *per se*, but also give rise to plasmacytoid dendritic cells which are considered as the primary type I IFN producing cells in SLE [11, 12].

Several powerful computational tools have facilitated *de novo* drug development and drug repurposing processes in a cost-effective and time-saving manner. The library of integrated network-based cellular signatures (LINCS) L1000 dataset integrated over a million gene expression profiles of human cell lines before and after exposure to more than 20,000 perturbagens. Taking a step forward, the LINCS L1000 Characteristic Direction Signatures Search engine (L1000CDS^2^) enabled the prioritization of thousands of small-molecule signatures, according to their ability to counteract disease specific transcriptional profiles [13]. We have previously employed an iLINCS-based drug repurposing pipeline [14, 15], suggesting the potential therapeutic relevance of compounds targeting the PI3K/mTOR pathway in SLE.

Herein, we employed two independent drug repurposing approaches to identify novel compounds that might restore the molecular aberrancies of monocytes in SLE. Using the iLINCS, CLUE and L1000CDS^2^ platforms, we propose putative novel drugs potentially capable of reversing the monocyte-related SLE gene signature. We also report FDA-approved drugs and patented compounds that might disturb the gene regulatory network of SLE monocytes, suggesting they should be tested as monocyte-targeted therapies in SLE.

## Results

### The SLE monocyte gene signature can be utilized to predict potential drug repurposing

To propose existing FDA-approved or investigational compounds that might serve as novel monocyte-targeted therapies in SLE, we sought to identify compounds with potency to reverse the monocyte gene expression profile. Differentially expressed genes (DEGs) (absolute Fold Change ≥ 1.5, P-value ≤ 0.01) of monocytes between SLE patients and healthy individuals defined the monocyte-specific signature (Supplementary Tables 1, Supplementary Fig. 1). Using the iLINCS, CLUE and L1000CDS^2^ platforms, the top 50 compounds that were predicted to counteract the SLE monocyte-specific gene signature most efficiently – according to their inhibitory scores – were identified (Supplementary Tables 2–4).

Our analysis indicated several p38 MAP kinase inhibitors, such as the “L-skepinone” [16], as a potential novel strategy of tuning monocytes in SLE. Additionally, the mTOR inhibitor “sirolimus” [17], as well as the calcineurin inhibitor “tacrolimus” [18], were recognized as potent modulators of the lupus monocyte gene signature. In line with studies underlying the crucial role of NF-κB in the survival and activation of monocytes [19], NF-κΒ pathway inhibitors, such as the compound “parthenolide” [20, 21], were predicted to reverse the SLE monocyte gene signature, whereas agents targeting the SLE-related Pim-1/NFATc1/NLRP3 signaling axis [22] might also represent promising therapeutic approaches. The sphingosine-1 phosphate receptor modulator “fingolimod”, which has shown possible efficacy in neuropsychiatric lupus manifestations in the MRL/*lpr* lupus mouse model [23], might therapeutically interfere with the monocyte-mediated orchestration of immune responses in SLE.

**Fig. 1:**
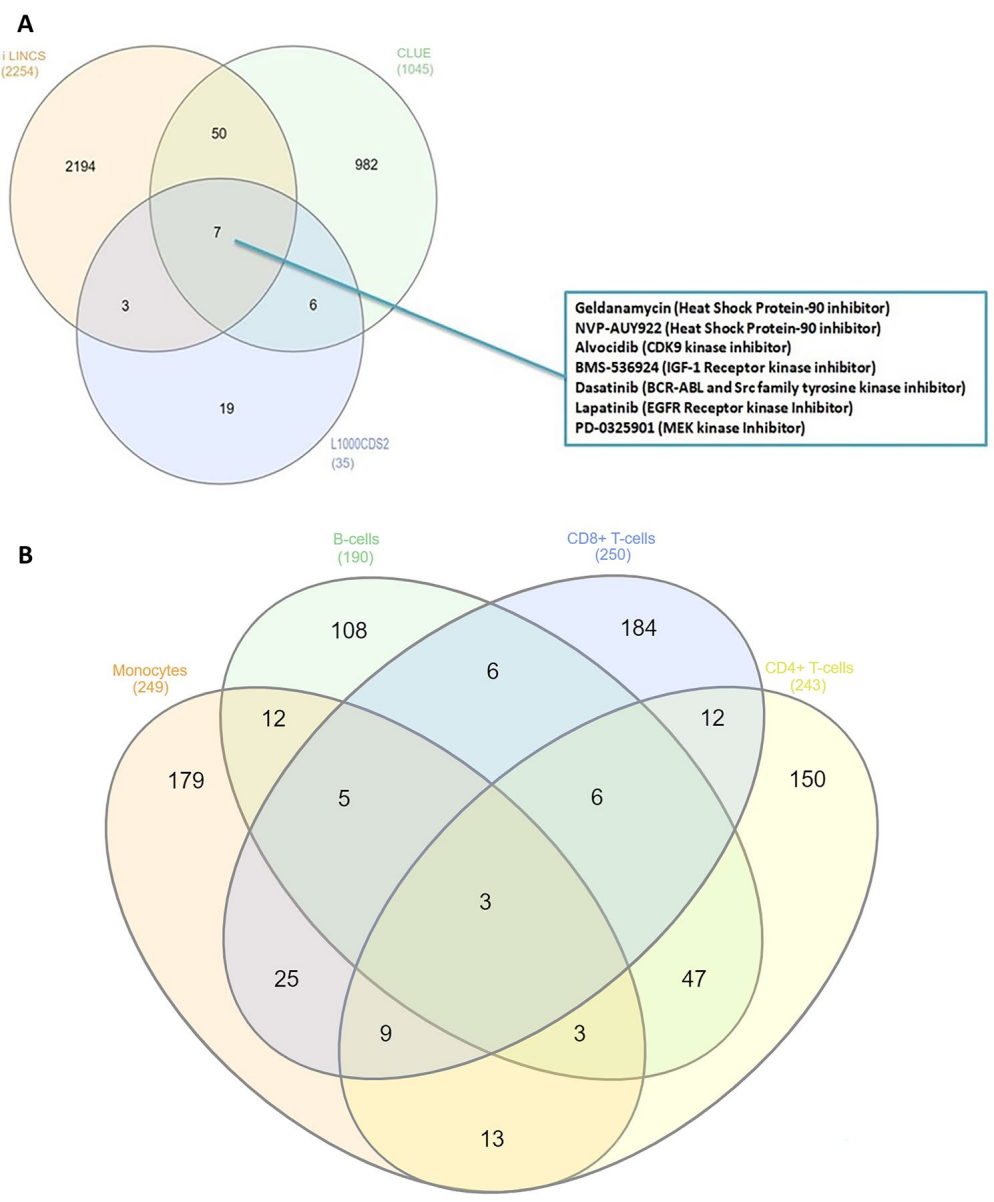
(**A**) Venn diagram demonstrating the common compounds identified by the three different drug repurposing platforms, iLINCS, CLUE and L1000CDS^2^, that could reverse the monocytes signature. To identify the 7 common compounds between the 3 different tools, all compounds derived from each different tool (iLINCS, CLUE and L1000), that could reverse the monocytes signature, were included in the Venn diagram. (**B**) Venn diagram to determine the top ranked drugs from all the three different drug repurposing platforms, that reverse exclusively our monocytes signature and not the signatures of B-cells, CD4^+^ and CD8^+^-T cells. For this Venn diagram the top-50 ranked compounds derived exclusively from each different tool (iLINCS, CLUE and L1000) that could reverse the monocytes, the B-cells, CD4^+^ and CD8^+^-T cells signature respectively, were included. Concerning the iLINCS tool the top-50 ranked compounds from each of the 5 different libraries were included. Conclusively, via this Venn diagram, 179 compounds were identified to reverse exclusively the monocytes signature

The common compounds reversing the monocyte gene signature were identified by the three different platforms (Fig. 1Α): the heat shock protein 90 inhibitors “geldanamycin” and “NVP-AUY922”, the Insulin-like growth factor 1 receptor (IGF-1R) inhibitor “BMS-536924”, the BCR-ABL and Src family tyrosine kinase receptor inhibitor “dasatinib”, the Cyclin-Dependent Kinase 9 inhibitor “alvocidib”, the EGFR inhibitor “lapatinib” and the MEK kinase inhibitor “PD-0325901.

Among the compounds identified by the L1000CDS^2^ that were highly ranked in the other 2 tools (CLUE and iLINCS) were the HSP90 inhibitors “geldanamycin” and “NVP-AUY922”, the EGFR inhibitors “gefitinib”, “lapatinib” and “canertinib” the DNA damage checkpoint kinase 1 and 2 (chk-1 and chk-2) inhibitor “AZD-7765”, the Insulin-like growth factor 1 receptor (IGF-1R) inhibitor “BMS-536924”, the Cyclin-Dependent Kinase 9 inhibitor “alvocidib”, the glucogen synthase inhibitor “TWS-119” and the Src/ABL dual kinase inhibitor “saracatinib”.

To enhance the specificity of our drug repurposing approach, leveraging the iLINCS, CLUE and L1000CDS^2^ platforms, we identified the 50 top-ranked compounds, that could reverse the publicly available transcriptional signatures of circulating CD4^+^ [56], CD8^+^-T [56] and B-cells [57] obtained from SLE patients and we determined the agents that were exclusively related to the monocytes (Fig. 1B). The NF-κΒ pathway inhibitor “parthenolide”, inhibitors of the Pim-1/NFATc1/NLRP3 signaling axis, the EGFR inhibitors “gefitinib” and “afatinib”, the spleen tyrosine kinase (SYK) inhibitor “fostamatinib”, the TGF-beta inhibitor “pirfenidone”, the dual Src/ABL kinase inhibitor saracatinib, the antioxidant “L-sulphoraphane” as well as the “AZD-7765”, a compound inhibiting the DNA damage checkpoint kinase chk-1 and chk-2 were identified as monocyte specific.

### Gene interaction network analysis as a guide for drug repurposing

**Fig. 2:**
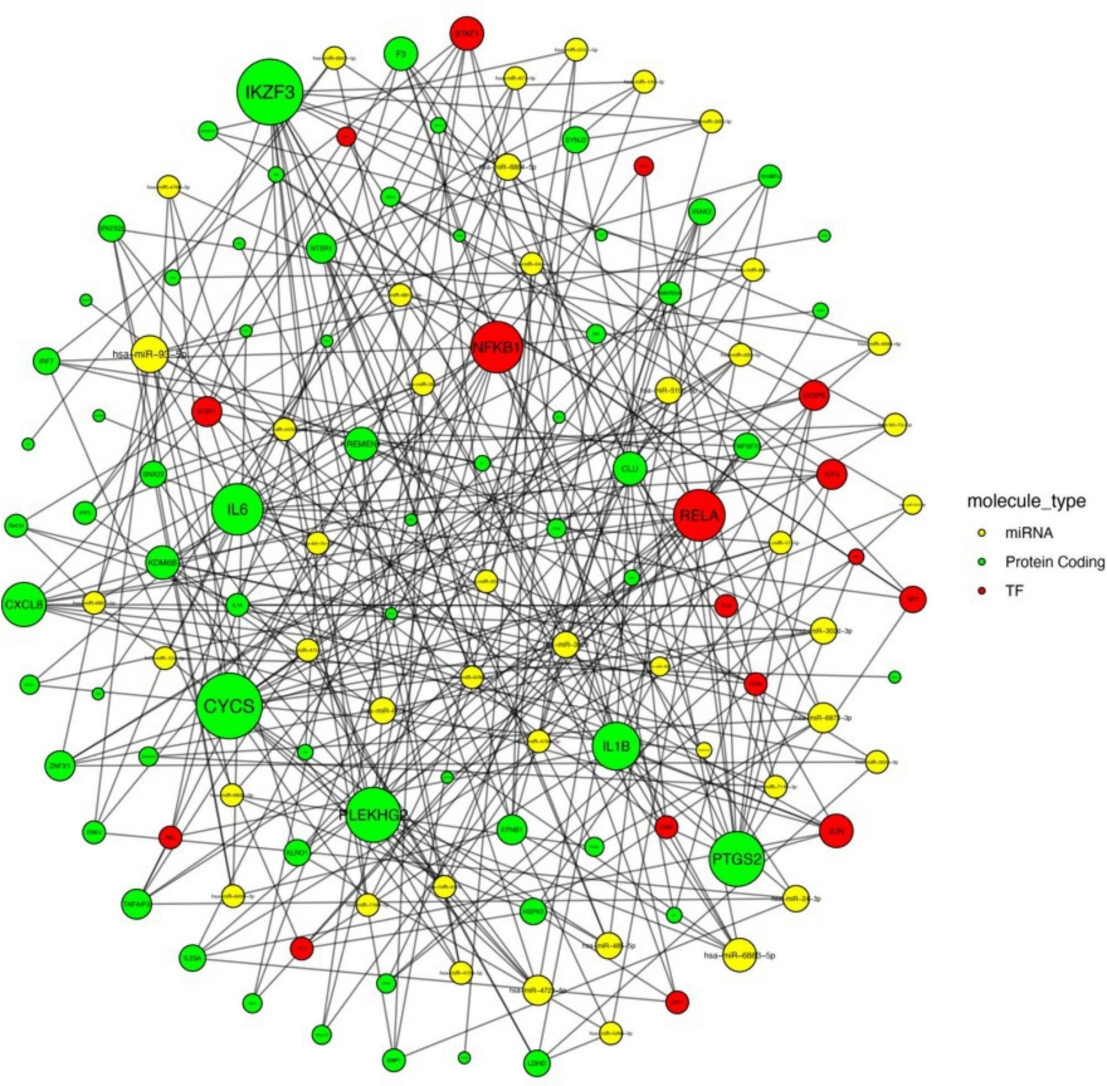
Interaction network integrating the protein coding differentially expressed genes (DEGs) identified by the differential expression analysis of the monocytes from SLE patients versus healthy individuals, the transcription factors identified to regulate their expression and the miRNAs that are associated with them. The size of each node is scaled according to the degree of its interconnectivity, with highly interconnected nodes depicted larger. Only nodes with degree > 3 were depicted. Genes encoding the interleukins IL-6, IL-1b as well as genes implicated in the JAK/STAT pathway were among the most highly interconnected nodes

Next, we sought to propose compounds that modulate the expression of multiple targets in the gene regulatory network of SLE monocytes. To this end, the transcription factors that regulate the transcriptional landscape of monocytes in SLE were retrieved from the TRRUST database (Supplementary Table 5). To reveal post-transcriptional regulators, the miRNAs that could regulate the gene expression profile of SLE monocytes were yielded using the miRWalk database (Supplementary Table 6). Thus, a comprehensive miRNA-gene interaction network - inferred using the monocytes gene signature, transcription factors and miRNAs - was constructed (Fig. 2).

Topological analysis of the constructed network uncovered a high degree of interconnectivity of genes encoding the proinflammatory mediators IL-6 and IL-1b. In line with studies underscoring the pivotal contribution of monocytes as IFN-producing cells in SLE, genes linked to type I IFN pathway, such as *IRF7*, *IFIT3*, as well as the transcription factor *STAT1* emerged as hub nodes [24]. Top-ranked hub miRNAs included the miR-124-3p, which has been found significantly upregulated in peripheral blood mononuclear cells and serum from SLE patients [25], as well as several miRNAs, with still largely unknown function in the context of SLE, such as miR-24-3p, miR-302c-3p and miR-302d-3p.

**Fig. 3:**
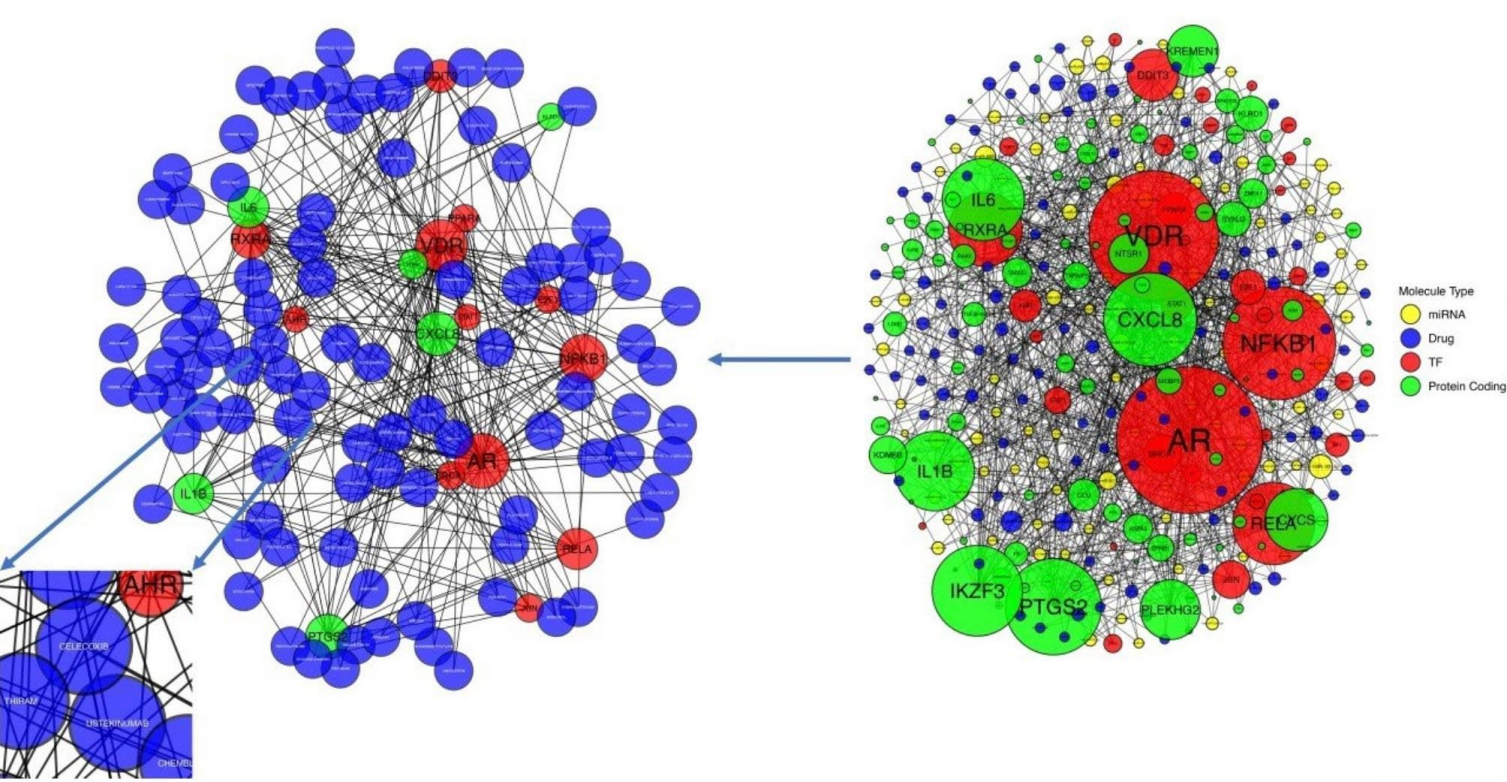
Interaction network combining the protein coding differentially expressed genes (DEGs), the transcription factors and the miRNAs as defined in Fig. 2 and the drugs that are predicted to interact with the DEGs, according to the DGIdb database. Nodes with degree > 2 were included in the network on the right side of the graph. From the nodes included in the network on the right side of the graph, we selected the DEGs, transcription factors and miRNAs with degree > 10, as depicted in the network on the left side of the graph. Among others, the monoclonal antibodies targeting the IL12/IL23 as well as the TNF pathways were identified

To identify agents with potentially unrecognized efficacy in SLE, we next determined drugs targeting hub genes of the miRNA-gene interaction network. Using the DGIdb database, a detailed drug-gene interaction network was constructed (Fig. 3A, Supplementary Table 7), revealing the anti-IL-12/IL-23 antibody “ustekinumab” and the epidermal growth factor receptor (EGFR) inhibitor “cetuximab”. Interestingly, the recombinant human TNF receptor Fc fusion protein “etanercept” as well as the chimeric monoclonal anti-TNFa antibody “infliximab” were identified as highly interconnected nodes.

**Fig. 4:**
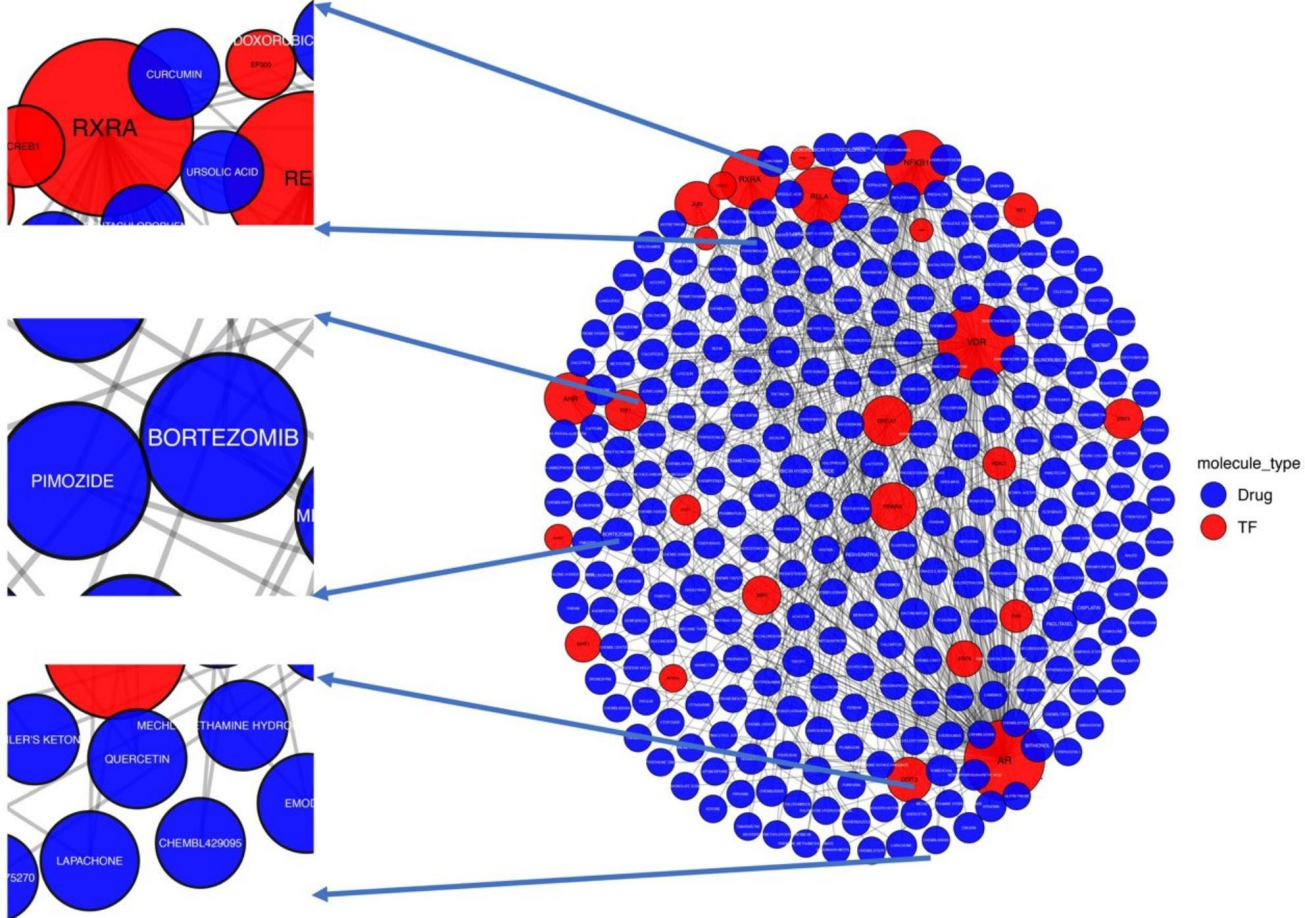
Interaction network showing the transcription factors that regulate the expression of the monocyte gene signature in SLE and the compounds that interfere with their function. Only nodes with degree > 2 were demonstrated. The proteasome inhibitor “bortezomib” as well as several natural products emerged as potential drug candidates

Considering the extensive alterations of transcriptional regulation in SLE monocytes, we additionally constructed the drug-transcription factor interaction network (Fig. 4). The proteasome inhibitor “bortezomib” was yielded as potential drug candidate, whereas several natural compounds and plant extracts, such as “resveratrol”, “quercetin” and “curcumin” might efficiently modulate the activity of the dysregulated transcription factors in SLE monocytes [26–30].

## Discussion

Herein, we applied a transcriptome-reversal combined with a network-based drug repurposing approach to identify novel compounds which might represent putative therapeutic options in SLE, through targeting transcriptional disturbances of monocytes. Using high-throughput drug repurposing tools, we identified agents predictive of reversing the molecular aberrations of SLE monocytes. By employing a gene network-based analysis, we propose agents, that might target essential regulators of the monocyte transcriptional landscape.

Several *in silico* drug repurposing studies have deployed whole blood gene expression profiling to suggest tailored SLE treatment choices [14, 31]. For example, Toro-Dominguez et al. employed a longitudinal stratification strategy to propose endotype tailored drug candidates [31]. In view of the central role of monocytes in several aspects of SLE pathogenesis [1], it is tempting to speculate that the targeted manipulation of monocytes might confer some therapeutic benefit in SLE. Furthermore, it was shown that selective depletion of monocytes in SLE patients by cytapheresis could lead to clinical remission, while inhibition of monocytes activation, differentiation and migration in in vivo models of SLE could have a beneficial impact on the disease manifestations such as nephritis and neuropsychiatric symptoms [53–55].To this end, we proposed putative novel drugs or small-molecule compounds that may reverse the transcriptional signatures of SLE monocytes. The inhibitor of the serine/threonine kinase Pim-1 “SGI-1776” was identified as a promising therapy, corroborating experimental data which suggest that inhibition of the Pim-1/NFATc1/NLRP3 pathway ameliorates nephritis in lupus mouse models [22]. Interestingly, the antioxidant “L-sulphoraphane” improved the renal damage in lupus-like mice through suppression of oxidative stress, enabling a mechanistic insight into our findings [49]. In the same context, the administration of “fostamatinib” in lupus prone mouse models prevented the development of nephritis, providing evidence for the therapeutic potential of targeting the Spleen tyrosine kinase (Syk) in SLE [47, 48, 61].

Serum IL-23 levels were significantly elevated in patients with SLE compared to healthy individuals and correlated with overall SLE disease activity as measured by SLEDAI [60]. Furthermore, the increased c-reactive protein levels observed in inflammatory conditions, especially during flares of the disease can be linked specifically with the IL-23 production in monocytes [58]. Despite the recently published phase 3 trial [33, 34], our findings indicate that the IL-12/IL-23 inhibitor “ustekinumab” may efficiently disrupt the molecular interaction network of monocytes and therefore some patients might indeed benefit from this drug. Although proteasome inhibitors effectively deplete autoreactive plasma cells and proved to be therapeutically effective in preclinical mouse models of LN, there is compelling evidence suggesting that immunoproteasome inhibition might selectively induces apoptosis in CD14 + monocytes, leading to suppression of IL-23-driven autoimmunity [52].

EGF is a chemoattractant for monocytes, implicated with the recruitment of monocytes at the sites of inflammation [50], whereas urine epidermal growth factor (EGF) levels might serve as a potential biomarker of response to treatment in patients with Lupus Nephritis (LN) [51]. Notably, monocytes are abundantly present in renal biopsies from patients with LN, therefore EGF receptor inhibitors, such as cetuximab might represent a potential new therapeutic avenue in LN through preventing abnormal migration of monocytes to LN inflammatory lesions.

Previous in vitro and in vivo data support the notion that HSP90 might represent a potential drug target in SLE [35–37]. Interestingly, HSP90 facilitates the TLR7/9-mediated nucleic acid recognition in SLE, therefore promoting IFN-α production from plasmacytoid dendritic cells [35]. To this end, the potential therapeutic application of the HSP90 inhibitor, “geldanamycin”, revealed by our analysis could merit further clinical investigation.

Complete understanding of miRNA regulation in SLE still remains elusive. Herein, we detected novel miRNAs, which might possess regulatory properties in the gene network of SLE monocytes. Given that each miRNA could concurrently influence multiple effectors of pathways, targeting the dysregulated miRNAs may also show promise for the future treatment of SLE. Accordingly, therapeutic modulation of the highly interconnected miR-124-3p and miR-302d, which has been designated as predictor of remission in SLE [25] and may participate in regulation of the IFN-induced gene expression in SLE through targeting the *IRF9* [59], respectively, might shed new insights into SLE treatment.

Our study has certain limitations, related to the function and topology of the cell subset and the methods used. Tissue macrophage compartment in steady state is mainly derived from embryonic precursors and actively contributes to maintenance of tissue homeostasis and resolution of inflammation [32]. Therefore, targeted pharmacological manipulation of tissue resident macrophage populations that might be driving pathology in SLE needs to be evaluated. Notably, the majority of the patients included in our study were receiving immunosuppressive treatment at sampling. Although cytotoxic agents, which can easily alter the composition of the whole-blood cells and potentially generate misleading results, were only rarely administrated in our study population, we can not exclude the possibility that therapy-induced transcriptional changes may interfere with our findings. Additionally, in our analysis we identified agents that might restore the transcriptional aberrations of SLE monocytes, however an effect of these agents on other cell types as well, cannot be completely excluded. Lastly, our analysis is a computational approach and further experimental and clinical investigation is required to validate our findings.

## Conclusion

In summary, using two independent computational system biology approaches, we identified novel compounds that are predicted to restore the function of monocytes in SLE. The therapeutic implications of our findings need to be further defined in animal models of SLE models and then tested in clinical trials.

## Methods

### Patients

Monocytes were isolated (CD14^+^ cells through FACS technology, BD FACS ARIA IIu) from peripheral blood samples of 15 SLE patients fulfilling the 2019 EULAR/ACR classification criteria for SLE [38]. Patients were recruited from the Rheumatology Outpatient Department of the Attikon University Hospital and the University Hospital of Heraklion [38] (supplementary Table 9). Ten age- and sex-matched healthy individuals were used as controls. Disease activity was evaluated using the modified Systemic Lupus Erythematosus Disease Activity Index 2000 (SLEDAI-2 K); SLEDAI-2 K ≥ 4 defined active disease [39, 40]. All participants provided informed consent and the study approval was obtained from the local institutional review boards.

### RNA sequencing and differential expression analysis

RNA libraries were prepared using the Illumina TruSeq kit. Paired-end mRNA sequencing was performed on the Illumina HiSeq2000 platform. The reads were aligned to the human reference genome (GRCh38.p12) by STAR RNA-Seq aligner [41]. Differential expression analysis was conducted using the edgeR Bioconductor R package [42].

### Drug repurposing analysis

Using the iLINCS [43], CLUE [44] and L1000CDS^2^ [13] platforms, we identified compounds that reverse the SLE monocyte signature. The following libraries were used for search in the iLINCS platform: (a) iLincs chemical perturbagen library (LINCSCP); (b) Connectivity map signatures library (CMAP); (c) Drug matrix signatures library (DM); (d) Cancer therapeutics response signatures library (CTRS); and (e) Pharmacogenomics transcriptional signatures library (PG). Through extensive literature review, the top-ranked compounds derived from each platform, were re-evaluated based on their functional relation to SLE-associated gene or protein targets (supplementary Fig. 2).

### Network analysis

The transcription factors and the microRNAs (miRNAs) that regulate the expression of the statistically significant, differentially expressed protein-coding genes were identified using the databases TRRUST and miRWalk, respectively. The drug-protein interactions were retrieved from the DGIdb database. Networks were constructed using the igraph package and their visualizations using the ggraph and qgraph packages in R [45, 46] (supplementary Fig. 3).

## Electronic supplementary material

Below is the link to the electronic supplementary material.

Supplementary **Table 1.** List of the statistically significant differentially expressed genes (DEGs) resulting from the comparison of the monocytes from SLE patients versus healthy controls.

Supplementary **Table 2.** List of Compounds that are predicted to reverse the monocytes signature in SLE, derived from the iLINCS platform-based drug repurposing analysis.

Supplementary **Table 3.** List of Compounds that are predicted to reverse the monocytes signature in SLE, derived from the CLUE platform-based drug repurposing analysis.

Supplementary **Table 4.** List of Compounds that are predicted to reverse the monocytes signature in SLE, derived from the L1000CDS2 platform-based drug repurposing analysis.

Supplementary **Table 5.** List of the transcription factors that are predicted to regulate the monocytes gene expression signature in SLE, as retrieved from the TRRUST database.

Supplementary **Table 6.** List of the miRNAs that are predicted to regulate the expression of the monocytes gene signature in SLE, as retrieved from the miRWalk database.

Supplementary **Table 7.** Topological analysis of the interaction network of Figure 3A

Supplementary **Table 8.** Full list of DEGs of monocytes, derived from the comparison of SLE patients versus healthy control individuals, without cut-off values.

Supplementary **Table 9.** Clinical parameters (including disease activity and concominant treatments) of the patients participated in the study. Abbreviations: MMF (mycophenolate-mofetil), AZA(azathioprine), GC(glucocorticoids), HCQ(hydroxychloroquine), CY(cyclophosphamide), ANA (anti-nuclear antibodies).

Supplementary **Table 10.** List of monocyte specific small molecule compounds, as identified via the iLINCS, CLUE io and L1000CDS2 platforms, in line with figure 1B.

Supplementary 11 **Figure 1.** Volcano plot, including the full list of DEGs from monocytes of SLE patients vs healthy controls.

Supplementary 12 **Figure 2.** Analysis methodology steps for the identification of small molecule compounds via the iLINCS, CLUE io and L1000CDS2.

Supplementary 13 **Figure 3.** Analysis methodology steps for the identification of small molecule compounds and biologic agents via the construction of Drug-Protein-miRNA interaction networks.

## Data Availability

All data generated or analyzed during this study are included in this published article [and its supplementary information files].

## List of abbreviations

SLE: Systemic lupus erythematosus
SLEDAI-2 K: Systemic Lupus Erythematosus Disease Activity Index 2000
miRNAs: MicroRNAs
HSP90: Heat shock protein 90
BLyS: B-lymphocyte stimulator
IFN: Interferon
LINCS: Library of integrated network-based cellular signatures
L1000CDS^2^: L1000 Characteristic Direction Signatures Search engine
DEGs: Differentially expressed genes
IGF-1R: Insulin-like growth factor 1 receptor
EGFR: Epidermal growth factor receptor
LN: Lupus Nephritis

## References

[1] Ma W-T, Gao F, Gu K, Chen D-K (2019). The role of Monocytes and Macrophages in Autoimmune Diseases: a Comprehensive Review. Front Immunol.

[2] Katsiari CG, Liossis SN, Souliotis VL, Dimopoulos AM, Manoussakis MN, Sfikakis PP (2002). Aberrant expression of the costimulatory molecule CD40 ligand on monocytes from patients with systemic lupus erythematosus. Clin Immunol.

[3] Harigai M, Hara M, Fukasawa C, Nakazawa S, Kawaguchi Y, Kamatani N (1999). Responsiveness of peripheral blood B cells to recombinant CD40 ligand in patients with systemic lupus erythematosus. Lupus.

[4] Higuchi T, Aiba Y, Nomura T, Matsuda J, Mochida K, Suzuki M (2002). Cutting edge: ectopic expression of CD40 ligand on B cells induces lupus-like autoimmune disease. J Immunol.

[5] Wang X, Huang W, Schiffer LE, Mihara M, Akkerman A, Hiromatsu K (2003). Effects of anti-CD154 treatment on B cells in murine systemic lupus erythematosus. Arthritis Rheum.

[6] Li Y, Lee PY, Reeves WH. (2010). Monocyte and macrophage abnormalities in systemic lupus erythematosus. Arch Immunol Ther Exp (Warsz). 2010 Oct;58(5):355 – 64. doi: 10.1007/s00005-010-0093-y. Epub 2010 Jul 31. PMID: 20676786; PMCID: PMC3785254. 10.1007/s00005-010-0093-y PMC3785254 20676786

[7] Herrmann M, Voll RE, Zoller OM, Hagenhofer M, Ponner BB, Kalden JR. (1998). Impaired phagocytosis of apoptotic cell material by monocyte-derived macrophages from patients with systemic lupus erythematosus. Arthritis Rheum. 41:1241–50. doi: 10.1002/1529-0131(199807)41:7<1241::AID-ART15>3.0.CO;2-H. 10.1002/1529-0131(199807)41:7<1241::AID-ART15>3.0.CO;2-H 9663482

[8] Kavai M, Szegedi G. (2007). Immune complex clearance by monocytes and macrophages in systemic lupus erythematosus. Autoimmun Rev. (2007) 6:497–502. doi: 10.1016/j.autrev.2007.01.017. 10.1016/j.autrev.2007.01.017 17643939

[9] Umare V, Pradhan V, Nadkar M, Rajadhyaksha A, Patwardhan M, Ghosh KK et al. (2014). Effect of proinflammatory cytokines (IL-6, TNF-alpha, and IL-1beta) on clinical manifestations in Indian SLE patients. Mediators Inflamm. 2014:385297. doi: 10.1155/2014/385297. 10.1155/2014/385297 PMC4273527 25548434

[10] Jin O, Sun LY, Zhou KX, Zhang XS, Feng XB, Mok MY (2005). Lymphocyte apoptosis and macrophage function: correlation with disease activity in systemic lupus erythematosus. Clin Rheumatol.

[11] Santer DM, Yoshio T, Minota S, Moller T, Elkon KB. (2009). Potent induction of IFN-alpha and chemokines by autoantibodies in the cerebrospinal fluid of patients with neuropsychiatric lupus. J Immunol. (2009) 182:1192–201. doi: 10.4049/jimmunol.182.2.1192. 10.4049/jimmunol.182.2.1192 PMC2745922 19124763

[12] Rönnblom L, Leonard D. (2016). Interferon pathway in SLE: one key to unlocking the mystery of the disease. Lupus Science & Medicine 2019;6:e000270. doi:10.1136/lupus-2018-000270. 10.1136/lupus-2018-000270 PMC6703304 31497305

[13] Duan Q, Reid SP, Clark NR, Wang Z, Fernandez NF, Rouillard AD, Readhead B, Tritsch SR, Hodos R, Hafner M et al. (2016). L1000CDS2: LINCS L1000 characteristic direction signatures search engine. NPJ Syst. Biol. Appl. 2016;2:16015. doi: 10.1038/npjsba.2016.15. 10.1038/npjsba.2016.15 PMC5389891 28413689

[14] Garantziotis P, Nikolakis D, Doumas S, Frangou E, Sentis G, Filia A, Fanouriakis A, Bertsias G, Boumpas DT (2022).

[15] Frangou E, Garantziotis P, Grigoriou M, Banos A, Nikolopoulos D, Pieta A, Doumas SA, Fanouriakis A, Hatzioannou A, Manolakou T, Alissafi T, Verginis P, Athanasiadis E, Dermitzakis E, Bertsias G, Filia A, Boumpas DT. (2022). Cross-species transcriptome analysis for early detection and specific therapeutic targeting of human lupus nephritis. Annals of the rheumatic diseases, annrheumdis-2021-222069. Advance online publication. 10.1136/annrheumdis-2021-222069 10.1136/annrheumdis-2021-222069 PMC9484391 35906002

[16] Jin N, Wang Q, Zhang X, Jiang D, Cheng H, Zhu K. (2011). The selective p38 mitogen-activated protein kinase inhibitor, SB203580, improves renal disease in MRL/lpr mouse model of systemic lupus. Int Immunopharmacol. 2011;11(9):1319–1326. doi:10.1016/j.intimp.2011.04.015. 10.1016/j.intimp.2011.04.015 21549858

[17] Fernandez D, Perl A. (2010). mTOR signaling: a central pathway to pathogenesis in systemic lupus erythematosus?. Discov Med. 2010;9(46):173–178. PMC3131182 20350481

[18] Rafael-Vidal C, Altabás I, Pérez N, Mourino RC, Pego-Reigosa JM, Garcia S. (2021). Calcineurin and Systemic Lupus Erythematosus: The Rationale for Using Calcineurin Inhibitors in the Treatment of Lupus Nephritis. Int J Mol Sci. 2021;22(3):1263. Published 2021 Jan 27. doi:10.3390/ijms22031263. 10.3390/ijms22031263 PMC7865978 33514066

[19] Liu T, Zhang L, Joo D (2017). NF-κB signaling in inflammation. Sig Transduct Target Ther.

[20] Darwish N, Sudha T, Godugu K, Bharali DJ, Elbaz O, El-Ghaffar H, Azmy E, Anber N, Mousa SA (2019). Novel targeted Nano-Parthenolide molecule against NF-kB in Acute myeloid leukemia. Molecules.

[21] Doerner JL, Wen J, Xia Y et al. (2015). TWEAK/Fn14 Signaling Involvement in the Pathogenesis of Cutaneous Disease in the MRL/lpr Model of Spontaneous Lupus. J Invest Dermatol. 2015;135(8):1986–1995. doi:10.1038/jid.2015.124. 10.1038/jid.2015.124 PMC4504782 25826425

[22] Fu R, Xia Y, Li M et al. (2019). Pim-1 as a Therapeutic Target in Lupus Nephritis. Arthritis Rheumatol. 2019;71(8):1308–1318. doi:10.1002/art.40863. 10.1002/art.40863 30791224

[23] Mike EV, Makinde HM, Der E et al. (2018). Neuropsychiatric Systemic Lupus Erythematosus Is Dependent on Sphingosine-1-Phosphate Signaling. Front Immunol. 2018;9:2189. Published 2018 Sep 26. doi:10.3389/fimmu.2018.02189. 10.3389/fimmu.2018.02189 PMC6168636 30319641

[24] Elkon KB, Stone VV. (2011). Type I interferon and systemic lupus erythematosus. J Interferon Cytokine Res. 2011;31(11):803–812. doi:10.1089/jir.2011.0045. 10.1089/jir.2011.0045 PMC3216059 21859344

[25] Yan L, Jiang L, Wang B, Hu Q, Deng S, Huang J, Sun X, Zhang Y, Feng L, Chen W. (2022). Novel microRNA biomarkers of systemic lupus erythematosus in plasma: miR-124-3p and miR-377-3p. Clin Biochem. 2022 May 19:S0009-9120(22)00133-3. doi: 10.1016/j.clinbiochem.2022.05.004. Epub ahead of print. PMID: 35598633. 10.1016/j.clinbiochem.2022.05.004 35598633

[26] Jhou JP, Chen SJ, Huang HY, Lin WW, Huang DY, Tzeng SJ. (2017). Upregulation of FcγRIIB by resveratrol via NF-κB activation reduces B-cell numbers and ameliorates lupus. Exp Mol Med. 2017;49(9):e381. Published 2017 Sep 29. doi:10.1038/emm.2017.144. 10.1038/emm.2017.144 PMC5628277 28960214

[27] Pannu N, Bhatnagar A. (2020). Combinatorial therapeutic effect of resveratrol and piperine on murine model of systemic lupus erythematosus. Inflammopharmacology.2020;28(2):401–424. doi:10.1007/s10787-019-00662-w. 10.1007/s10787-019-00662-w 31732838

[28] Voloshyna I, Teboul I, Littlefield MJ et al. (2016). Resveratrol counters systemic lupus erythematosus-associated atherogenicity by normalizing cholesterol efflux. Exp Biol Med (Maywood). 2016;241(14):1611–1619. doi:10.1177/1535370216647181. 10.1177/1535370216647181 PMC4994911 27190277

[29] Alexander T, Sarfert R, Klotsche J, Kühl AA, Rubbert-Roth A, Lorenz HM et al. (2014). The Proteasome Inhibitior Bortezomib Depletes Plasma Cells and Ameliorates Clinical Manifestations of Refractory Systemic Lupus Erythematosus. Ann Rheum Dis (2015) 74(7):1474–8. doi: 10.1136/annrheumdis-2014-206016. 10.1136/annrheumdis-2014-206016 PMC4484251 25710470

[30] Li W, Li H, Zhang M et al. (2016). Quercitrin ameliorates the development of systemic lupus erythematosus-like disease in a chronic graft-versus-host murine model. Am J Physiol Renal Physiol. 2016;311(1):F217-F226. doi:10.1152/ajprenal.00249.2015. 10.1152/ajprenal.00249.2015 26911849

[31] Toro-Domínguez D, Lopez-Domínguez R, GarcíaMoreno A, Villatoro-García JA, Martorell-Marugán J, Goldman D, Petri M, Wojdyla D, Pons-Estel BA, Isenberg D, Morales-Montes de Oca G, Trejo-Zambrano MI, GarcíaGonzález B, Rosetti F, Gómez-Martín D, Romero-Díaz J, Carmona-Sáez P, Alarcón-Riquelme ME. (2019). Differential Treatments Based on Drug-induced Gene Expression Signatures and Longitudinal Systemic Lupus Erythematosus Stratification. SciRep. 2019 Oct 29;9(1):15502. doi: 10.1038/s41598-019-51616-9. PMID: 31664045; PMCID: PMC6820741. 10.1038/s41598-019-51616-9 PMC6820741 31664045

[32] Ginhoux F, Jung S (2014). Monocytes and macrophages: developmental pathways and tissue homeostasis. Nat Rev Immunol.

[33] van Vollenhoven RF, Hahn BH, Tsokos GC, Wagner CL, Lipsky P, Touma Z, Werth VP, Gordon RM, Zhou B, Hsu B, Chevrier M, Triebel M, Jordan JL, Rose S. (2018). Efficacy and safety of ustekinumab, an IL-12 and IL-23 inhibitor, in patients with active systemic lupus erythematosus: results of a multicentre, double-blind, phase 2, randomised, controlled study. Lancet. 2018 Oct 13;392(10155):1330–1339. doi: 10.1016/S0140-6736(18)32167-6. Epub 2018 Sep 21. PMID: 30249507. 10.1016/S0140-6736(18)32167-6 30249507

[34] van Vollenhoven RF, Kalunian KC, Dörner T, Hahn BH, Tanaka Y, Gordon RM, Shu C, Fei K, Gao S, Seridi L, Gallagher P, Lo KH, Berry P, Zuraw QC. (2022). Phase 3, multicentre, randomised, placebo-controlled study evaluating the efficacy and safety of ustekinumab in patients with systemic lupus erythematosus. Annals of the rheumatic diseases, annrheumdis-2022-222858. Advance online publication. 10.1136/ard-2022-222858. 10.1136/ard-2022-222858 PMC9606504 35798534

[35] Saito K, Kukita K, Kutomi G, Okuya K, Asanuma H, Tabeya T et al. (2015). Heat shock protein 90 associates with Toll-like receptors 7/9 and mediates self-nucleic acid recognition in SLE. Eur J Immunol. 2015;45:2028–41. 10.1002/eji.201445293 25871979

[36] Shimp S, Chafin C, Regna N (2012). Heat shock protein 90 inhibition by 17-DMAG lessens disease in the MRL/lpr mouse model of systemic lupus erythematosus. Cell Mol Immunol.

[37] Liu Y, Ye J, Shin Ogawa L, Inoue T, Huang Q, Chu J (2015). The HSP90 inhibitor Ganetespib alleviates Disease Progression and augments intermittent Cyclophosphamide Therapy in the MRL/lpr mouse model of systemic Lupus Erythematosus. PLoS ONE.

[38] Aringer M, Costenbader K, Daikh D, Brinks R, Mosca M, Ramsey-Goldman R, Smolen JS, Wofsy D, Boumpas DT, Kamen DL, Jayne D, Cervera R, Costedoat-Chalumeau N, Diamond B, Gladman DD, Hahn B, Hiepe F, Jacobsen S, Khanna D, Lerstrøm K, Massarotti E, McCune J, Ruiz-Irastorza G, Sanchez-Guerrero J, Schneider M, Urowitz M, Bertsias G, Hoyer BF, Leuchten N, Tani C, Tedeschi SK, Touma Z, Schmajuk G, Anic B, Assan F, Chan TM, Clarke AE, Crow MK, Czirják L, Doria A, Graninger W, Halda-Kiss B, Hasni S, Izmirly PM, Jung M, Kumánovics G, Mariette X, Padjen I, Pego-Reigosa JM, Romero-Diaz J, Rúa-Figueroa Fernández Í, Seror R, Stummvoll GH, Tanaka Y, Tektonidou MG, Vasconcelos C, Vital EM, Wallace DJ, Yavuz S, Meroni PL, Fritzler MJ, Naden R, Dörner T, Johnson SR. (2019). European League Against Rheumatism/American College of Rheumatology Classification Criteria for Systemic Lupus Erythematosus. Arthritis Rheumatol. 2019 Sep;71(9):1400–1412. doi: 10.1002/art.40930. Epub 2019 Aug 6. PMID: 31385462; PMCID: PMC6827566. 10.1002/art.40930 PMC6827566 31385462

[39] Yee CS, Farewell VT, Isenberg DA, Griffiths B, Teh LS, Bruce IN, Ahmad Y, Rahman A, Prabu A, Akil M, McHugh N, Edwards C, D’Cruz D, Khamashta MA, Gordon C. (2010). The use of Systemic Lupus Erythematosus Disease Activity Index-2000 to define active disease and minimal clinically meaningful change based on data from a large cohort of systemic lupus erythematosus patients. Rheumatology (Oxford). 2011 May;50(5):982-8. doi: 10.1093/rheumatology/keq376. Epub 2011 Jan 18. PMID: 21245073; PMCID: PMC3077910. 10.1093/rheumatology/keq376 PMC3077910 21245073

[40] Lam GKW, Petri M. (2005). Assessment of systemic lupus erythematosus. Clin Exp Rheumatol 2005;23(5 Suppl 39):S120–32. 16273796

[41] Dobin A, Davis CA, Schlesinger F, Drenkow J, Zaleski C, Jha S, Batut P, Chaisson M, Gingeras TR (2013). STAR: ultrafast universal RNA-seq aligner. Bioinf (Oxford England).

[42] Robinson MD, McCarthy DJ, Smyth GK (2010). edgeR: a Bioconductor package for differential expression analysis of digital gene expression data. Bioinformatics.

[43] Keenan AB, Jenkins SL, Jagodnik KM et al. (2018). The Library of Integrated Network-Based Cellular Signatures NIH Program: System-Level Cataloging of Human Cells Response to Perturbations. Cell Syst. 2018;6(1):13–24. doi:10.1016/j.cels.2017.11.001. 10.1016/j.cels.2017.11.001 PMC5799026 29199020

[44] Lamb J (2006). The Connectivity Map: using gene-expression signatures to connect small molecules, genes, and disease.

[45] Csardi G, Nepusz T. (2006). The igraph software package for complex network research.InterJournal, Complex Systems,1695. https://igraph.org.

[46] Epskamp S, Cramer AOJ, Waldorp LJ, Schmittmann VD, Borsboom D (2012). qgraph: network visualizations of Relationships in Psychometric Data. J Stat Softw.

[47] Bahjat FR, Pine PR, Reitsma A, Cassafer G, Baluom M, Grillo S, Chang B, Zhao FF, Payan DG, Grossbard EB, Daikh DI. (2008). An orally bioavailable spleen tyrosine kinase inhibitor delays disease progression and prolongs survival in murine lupus. Arthritis Rheum. 2008 May;58(5):1433-44. doi: 10.1002/art.23428. PMID: 18438845. 10.1002/art.23428 18438845

[48] Deng GM, Liu L, Bahjat FR, Pine PR, Tsokos GC. (2010). Suppression of skin and kidney disease by inhibition of spleen tyrosine kinase in lupus-prone mice. Arthritis Rheum. 2010 Jul;62(7):2086-92. doi: 10.1002/art.27452. PMID: 20222110; PMCID: PMC2902591. 10.1002/art.27452 PMC2902591 20222110

[49] Du P, Zhang W, Cui H, He W, Lu S, Jia S, Zhao M. Sulforaphane Ameliorates the Severity of Psoriasis and SLE by Modulating Effector Cells and Reducing Oxidative Stress.Front Pharmacol. 2022 Jan21;13:805508. doi: 10.3389/fphar.2022.805508. PMID: 35126161; PMCID: PMC8814458. 10.3389/fphar.2022.805508 PMC8814458 35126161

[50] Berrahmoune H, Lamont JV, Herbeth B, FitzGerald PS, Visvikis-Siest S. (2006). Biological determinants of and reference values for plasma interleukin-8, monocyte chemoattractant protein-1, epidermal growth factor, and vascular endothelial growth factor: Results from the STANISLAS cohort. Clin Chem. 2006 Mar;52(3):504 – 10. doi: 10.1373/clinchem.2005.055798. Epub 2006 Jan 19. PMID: 16423909. 10.1373/clinchem.2005.055798 16423909

[51] Ngamjanyaporn P, Worawichawong S, Pisitkun P, Khiewngam K, Kantachuvesiri S, Nongnuch A, Assanatham M, Sathirapongsasuti N, Kitiyakara C (2022). Predicting treatment response and clinicopathological findings in lupus nephritis with urine epidermal growth factor, monocyte chemoattractant protein-1 or their ratios. PLoS One 2022 Mar.

[52] Basler M, Claus M, Klawitter M, Goebel H, Groettrup M. Immunoproteasome Inhibition Selectively Kills Human CD14^+^ Monocytes and as a Result Dampens IL-23 Secretion. J Immunol. 2019 Oct 1;203(7):1776–1785. doi: 10.4049/jimmunol.1900182. Epub 2019 Sep 4. PMID: 31484727. 10.4049/jimmunol.1900182 31484727

[53] Soerensen H, Schneidewind-Mueller JM, Lange D, Kashiwagi N, Franz M, Yokoyama T, Ramlow W. (2006). Pilot clinical study of Adacolumn cytapheresis in patients with systemic lupus erythematosus. Rheumatol Int. 2006 Mar;26(5):409 – 15. doi: 10.1007/s00296-005-0031-1. Epub 2005 Sep 28. PMID: 16189656. 10.1007/s00296-005-0031-1 16189656

[54] Shimizu S, Nakashima H, Masutani K, Inoue Y, Miyake K, Akahoshi M, Tanaka Y, Egashira K, Hirakata H, Otsuka T, Harada M. (2004). Anti-monocyte chemoattractant protein-1 gene therapy attenuates nephritis in MRL/lpr mice. Rheumatology (Oxford). 2004 Sep;43(9):1121-8. doi: 10.1093/rheumatology/keh277. Epub 2004 Jun 22. PMID: 15213333. 10.1093/rheumatology/keh277 15213333

[55] Chalmers SA, Wen J, Shum J, Doerner J, Herlitz L, Putterman C. CSF-1R inhibition attenuates renal and neuropsychiatric disease in murine lupus. Clin Immunol. 2017 Dec;185:100–8. 10.1016/j.clim.2016.08.019. Epub 2016 Aug 26. PMID: 27570219; PMCID: PMC5326697. 10.1016/j.clim.2016.08.019 PMC5326697 27570219

[56] Buang N, Tapeng L, Gray V, Sardini A, Whilding C, Lightstone L, Cairns TD, Pickering MC, Behmoaras J, Ling GS, Botto M. Type I interferons affect the metabolic fitness of CD8 + T cells from patients with systemic lupus erythematosus. Nat Commun. 2021 Mar 31;12(1):1980. doi: 10.1038/s41467-021-22312-y. PMID: 33790300; PMCID: PMC8012390. 10.1038/s41467-021-22312-y PMC8012390 33790300

[57] Panwar B, Schmiedel BJ, Liang S, White B, Rodriguez E, Kalunian K, McKnight AJ, Soloff R, Seumois G, Vijayanand P, Ay F. Multi-cell type gene coexpression network analysis reveals coordinated interferon response and cross-cell type correlations in systemic lupus erythematosus.Genome Res. 2021Apr;31(4):659–676. doi: 10.1101/gr.265249.120. Epub 2021 Mar 5. PMID: 33674349; PMCID: PMC8015858. 10.1101/gr.265249.120 PMC8015858 33674349

[58] Geyer CE, Newling M, Sritharan L, Griffith GR, Chen HJ, Baeten DLP, den Dunnen J. C-Reactive Protein Controls IL-23 Production by Human Monocytes.Int J Mol Sci. 2021 Oct28;22(21):11638. doi: 10.3390/ijms222111638. PMID: 34769069; PMCID: PMC8583945. 10.3390/ijms222111638 PMC8583945 34769069

[59] Smith S, Fernando T, Wu PW, Seo J, Ní Gabhann J, Piskareva O, McCarthy E, Howard D, O’Connell P, Conway R, Gallagher P, Molloy E, Stallings RL, Kearns G, Forbess L, Ishimori M, Venuturupalli S, Wallace D, Weisman M, Jefferies CA. MicroRNA-302d targets IRF9 to regulate the IFN-induced gene expression in SLE. J Autoimmun. 2017 May;79:105–11. Epub 2017 Mar 17. PMID: 28318807. 10.1016/j.jaut.2017.03.003 28318807

[60] Xiong DK, Shi X, Han MM, Zhang XM, Wu NN, Sheng XY, Wang JN. The regulatory mechanism and potential application of IL-23 in autoimmune diseases.Front Pharmacol. 2022 Sep13;13:982238. doi: 10.3389/fphar.2022.982238. PMID: 36176425; PMCID: PMC9514453. 10.3389/fphar.2022.982238 PMC9514453 36176425

[61] Pohlmeyer CW, Shang C, Han P, Cui ZH, Jones RM, Clarke AS, Murray BP, Lopez DA, Newstrom DW, Inzunza MD, Matzkies FG, Currie KS, Di Paolo JA. (2021). Characterization of the mechanism of action of lanraplenib, a novel spleen tyrosine kinase inhibitor, in models of lupus nephritis. BMC Rheumatol. 2021 Mar 30;5(1):15. doi: 10.1186/s41927-021-00178-3. PMID: 33781343; PMCID: PMC8008554. 10.1186/s41927-021-00178-3 PMC8008554 33781343

