# Supplementary figures and images for: Restoration of aberrant gene expression of monocytes in systemic lupus erythematosus via a combined transcriptome-reversal and network-based drug repurposing strategy
Source: BMC Genomics. 2023 Apr 18;24:207. doi: 10.1186/s12864-023-09275-8 (PMC10114456; doi:10.1186/s12864-023-09275-8)

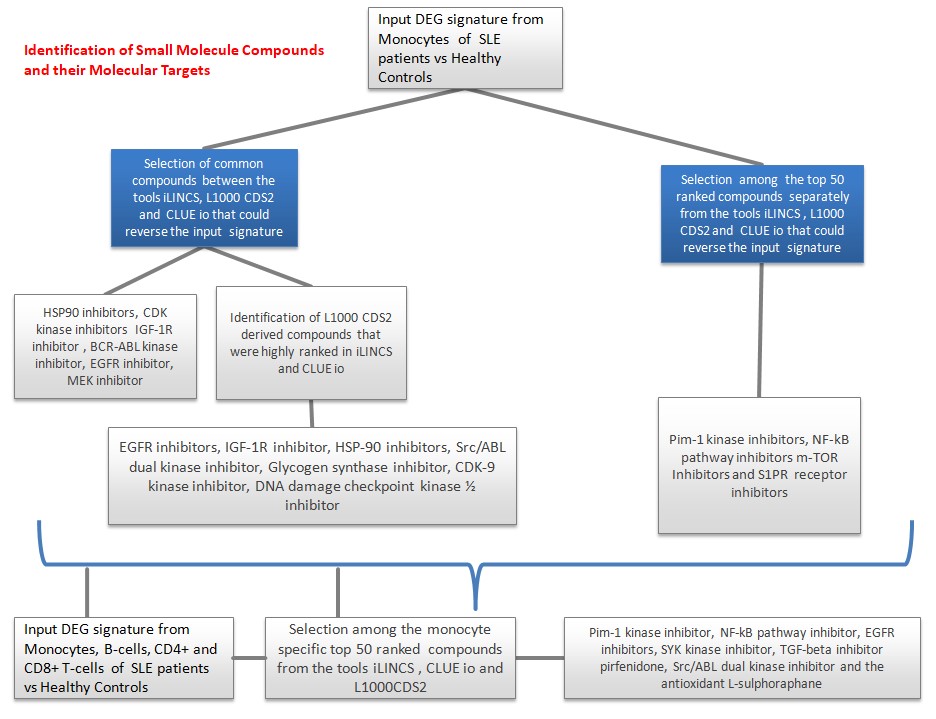

Supplement: Supplementary file 11 — Supplementary 11 Figure 1. Volcano plot, including the full list of DEGs from monocytes of SLE patients vs healthy controls. [file 12864_2023_9275_MOESM11_ESM.jpg]

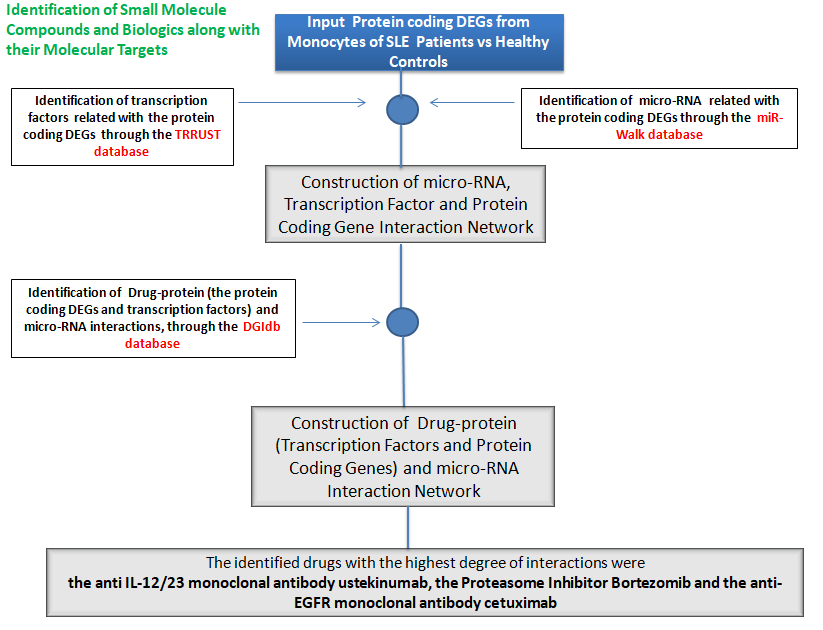

Supplement: Supplementary file 12 — Supplementary 12 Figure 2. Analysis methodology steps for the identification of small molecule compounds via the iLINCS, CLUE io and L1000CDS2. [file 12864_2023_9275_MOESM12_ESM.png]

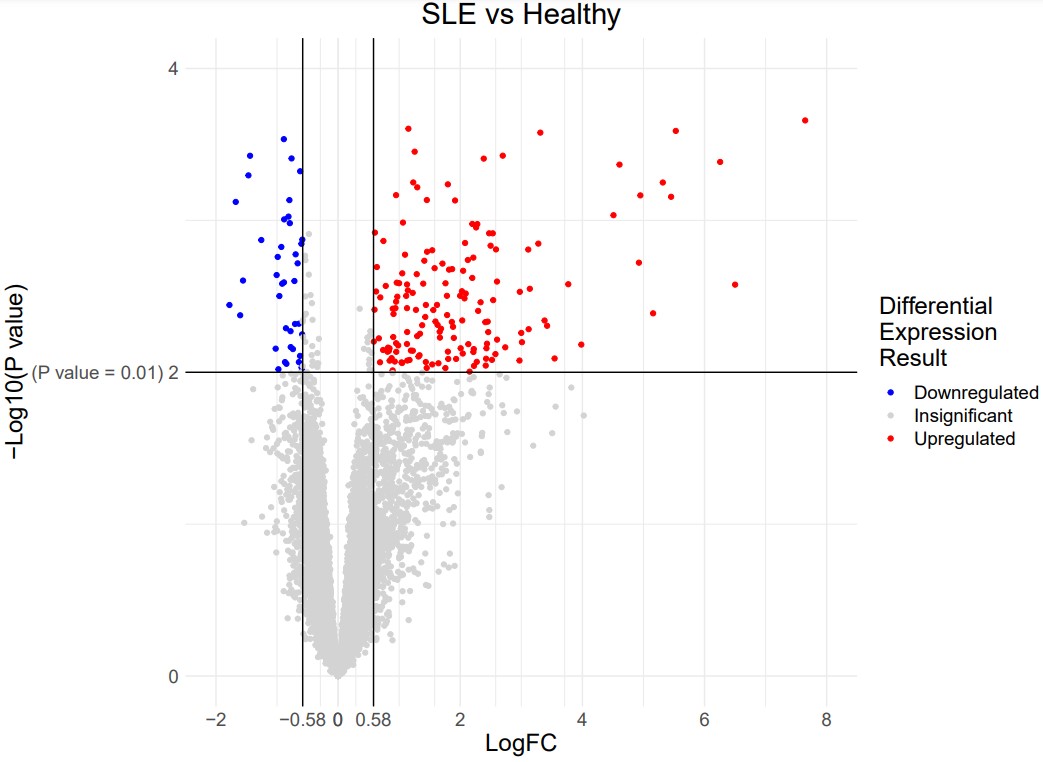

Supplement: Supplementary file 13 — Supplementary 13 Figure 3. Analysis methodology steps for the identification of small molecule compounds and biologic agents via the construction of Drug-Protein-miRNA interaction networks. [file 12864_2023_9275_MOESM13_ESM.jpg]
